# No Differential Reward Responsivity and Drive, Punishment Sensitivity or Attention for Cues Signaling Reward or Punishment in Adolescents With Obesity

**DOI:** 10.3389/fpsyg.2019.02363

**Published:** 2019-10-22

**Authors:** Nienke C. Jonker, Eva van Malderen, Klaske A. Glashouwer, Leentje Vervoort, Caroline Braet, Lien Goossens, Peter J. de Jong

**Affiliations:** ^1^Department of Clinical Psychology and Experimental Psychopathology, University of Groningen, Groningen, Netherlands; ^2^Clinical Developmental Psychology, Ghent University, Ghent, Belgium; ^3^Department of Eating Disorders, Accare Child and Adolescent Psychiatry, Groningen, Netherlands

**Keywords:** obesity, reward sensitivity, punishment sensitivity, attentional bias, adolescents

## Abstract

Although it has been proposed that obese and healthy weight individuals might differ in their reward and punishment sensitivity, the literature shows diverse and inconsistent findings. The current study was set out to examine the role of reward and punishment sensitivity in adolescent obesity by differentiating between reward responsivity and reward drive, and by complementing self-report measures with performance-based measures indexing attention for cues signaling reward and punishment as well as effort to approach reward and avoid punishment. Participants were adolescents aged 12–23, with obesity (*n* = 51, adjusted BMI [(actual BMI/Percentile 50 of BMI for age and gender) × 100) between 143 and 313%], and with a healthy weight (*n* = 51, adjusted BMI between 75 and 129%). Individuals with obesity did not significantly differ from adolescents with a healthy weight in reward responsivity, reward drive or attention to cues signaling reward. Further, no differences in self-reported punishment sensitivity or attention for cues signaling punishment were found between obese and healthy weight adolescents. The current study thus does not corroborate the theories that general reward and punishment sensitivity play a role in obesity.

## Introduction

The prevalence of obesity has nearly tripled worldwide since 1975 and currently 23.3% of the adult European population is obese (World Health Organization [WHO], 2016, 2018). Obesity is related to an increased risk of developing several chronic diseases (e.g., diabetes), psychological problems (e.g., anxiety and depression), and a lower life satisfaction (Dixon, 2010; Luppino et al., 2010; Roberts and Hao, 2013). Currently, 8.6% of European adolescents is obese (World Health Organization [WHO], 2016). Even though the prevalence of obesity in adolescents is lower than in adults, more than 80% of obese adolescents will become obese adults (Reilly et al., 2003). Adolescence thus seems an important period to intervene on obesity, with multidisciplinary interventions, combining diet, physical activity and behavioral lifestyle interventions as the treatment of choice (Stegenga et al., 2014). However, these interventions are limited in their effectiveness, suffer from high drop-out rates, and relapse after weight loss is common (Wilson, 1996; Poston et al., 1999; Goossens et al., 2009; Al-Khudairy et al., 2017). In order to increase the success rate of interventions and decrease the chances of relapse after weight loss, it is important to improve our understanding of the underlying factors of the development and maintenance of obesity. In the current study, we focused on individual differences in trait reward sensitivity, as this personality characteristic has been proposed to play an important role in eating behavior (e.g., De Decker et al., 2016), eating related disorders (e.g., Harrison et al., 2010; Matton et al., 2015), and obesity (e.g., Verbeken et al., 2012).

Individuals who are sensitive to reward are thought to attend more to cues of reward, respond more positively to reward, and show more approach behavior in response to reward in the environment (Gray, 1970; Gray and McNaughton, 2000). It was suggested that individuals who are sensitive to reward in general would also be more sensitive to the rewarding properties of food (Davis and Fox, 2008; i.e., the hyper-responsiveness model; Davis et al., 2007), and have an increased risk for overeating (Guerrieri et al., 2008). Furthermore, general reward sensitivity has been suggested to lead to more impulsive responses (e.g., Michaud et al., 2017), and impulsivity has also been implicated in the development and maintenance of obesity (e.g., Emery and Levine, 2017). As such, high general reward sensitivity might play a role in the development and maintenance of overweight and obesity. In apparent contrast to this, it has also been argued that a lowered general sensitivity to reward might be related to the development of obesity (e.g., Volkow et al., 2002; Wang et al., 2004). According to this view, also referred to as the *reward deficiency syndrome* theory (RDS), individuals try to compensate for reduced feelings of reward in general by eating large quantities of highly palatable foods (i.e., overeating). Last, the *dynamic vulnerability model* suggest a more dynamic relationship between general reward sensitivity and BMI. In this theory, high reward sensitivity is suggested to cause initial overeating and the development of overweight, as a consequence of overeating the reward response is suggested to decrease, thereby leading to more overeating in an effort to obtain the same rewarding feeling (e.g., Stice et al., 2011). If such a general trait might indeed be related to the development and or maintenance of obesity, this might warrant attention during treatment. However, although several studies have examined this relationship, findings have been inconsistent.

One approach that has been taken to examine whether and how reward sensitivity is related to obesity is by examining the continuous relationship between reward sensitivity and body mass index (BMI). Outcomes of this type of research have been mixed, ranging from no relationship between BMI and reward sensitivity (Matton et al., 2013; Jonker et al., 2016; Vandeweghe et al., 2017), to a positive relationship (Franken and Muris, 2005; De Decker et al., 2016), or a quadratic relationship (Davis and Fox, 2008; Verbeken et al., 2012; Dietrich et al., 2014). Also, a recent meta-analyses showed no significant relationship between reward sensitivity and BMI (Emery and Levine, 2017). As a second approach, research has compared individuals with a healthy weight and individuals with obesity with regard to their level of self-reported reward sensitivity. This might be a more appropriate approach than continuous modeling since the theories on the role of reward and punishment sensitivity are opposing each other, and suggest that there might be a non-linear relationship. One study showed that obese children were more sensitive to reward compared to healthy weight or overweight children, as reported by their parents (Van den Berg et al., 2011). However, other studies found no differences in reward sensitivity as reported by obese and healthy weight children (Nederkoorn et al., 2006), or adults (Schienle et al., 2009; Danner et al., 2012). Thus although there is some indication that reward sensitivity might be related to obesity, empirical findings so far are mixed.

An important reason for these inconsistency might be that these studies have measured different aspects of reward sensitivity. That is, in these studies, reward sensitivity has been indexed with the Behavioral Inhibition Scale/Behavioral Activation Scale (BIS/BAS; Carver and White, 1994) or the Sensitivity to Punishment and Sensitivity to Reward Questionnaire (SPSRQ; Torrubia et al., 2001; Colder and O’Connor, 2004). Although these questionnaires have been used interchangeably, they seem to index different aspects of reward sensitivity. The SPSRQ has one reward subscale that consists of a mixture of questions regarding reward responsivity and reward drive. The BIS/BAS consists of three subscales: reward responsivity, reward drive, and fun seeking, which are often averaged into a total reward sensitivity score. The fun seeking subscale of the BIS/BAS has, however, has been suggested to be a measure of impulsivity rather than of reward sensitivity (Carver and White, 1994; Scheres and Sanfey, 2006). Thus, this subscale and the total average scale of the BAS, in which it is included, might not be the most appropriate measures of reward sensitivity. Further, since reward responsivity and reward drive represent separate constructs that may be differentially involved in obesity, it seems critical to differentiate between these components for a proper appreciation of the possible role of reward sensitivity in obesity.

None of the studies that examined the role of reward drive in obesity by comparing obese and healthy weight individuals examined reward drive and responsivity separately. Although there are three studies that examined the relationship between reward drive and BMI, these studies show inconsistent results. One study showed no relationship (Jonker et al., 2016), one a positive relationship (De Decker et al., 2016), and one a quadratic relationship (Verbeken et al., 2012) between reward drive and BMI. Only one study has previously examined the role of reward responsivity in relation to BMI, yet failed to find a relationship (Jonker et al., 2016). Since in the sample of De Decker et al. (2016) and Michels et al. (2012) only 0.4%, and in the sample of Jonker et al. (2016) only 2.7% of the adolescents were obese, it remains unclear what the role of these constructs is in obesity. Thus, all in all, there is not only inconsistency in the outcomes of studies on the role of reward sensitivity in obesity, but also inconsistency in the aspect of reward sensitivity that is measured, and several studies included only few obese individuals in their sample. Therefore, the current study examined differences between a group of obese adolescents and a group of healthy weight adolescents with regard to both reward responsivity and reward drive. Although such a group comparison will not provide insight into whether reward sensitivity is a pre- or post-obesity characteristic, it is an important first step to establish whether obese adolescents actually differ from healthy weight adolescents.

Finally, although current theories about reinforcement sensitivity imply that individual differences in reward sensitivity can also be reflected in individual differences in individuals’ proneness to detect cues predicting reward (Gray, 1970; Gray and McNaughton, 2000; Davis and Fox, 2008), this component of reinforcement sensitivity has not yet been assessed in the context of obesity. To get a more comprehensive view on how reward sensitivity might be involved in obesity, this study therefore complemented the self-report measures of reward sensitivity with a performance-based measure that can index individuals’ proneness to detect signals of reward. More specifically, we relied on the Spatial Orientation Task (SOT; Derryberry and Reed, 2002) to examine whether adolescents with obesity would show relatively strong attentional bias for cues signaling reward.

Previously, in a large sample of adolescents (*N* = 610), no relationship was found between attention to cues signaling reward as measured with the SOT and BMI, or the change in BMI over 6 years (Jonker et al., 2016). However, since in this sample only 2.7% of the adolescents were obese, the role of attention to cues signaling reward in obese vs. healthy weight adolescents could not be properly examined. Therefore, the present study examined group differences in attention for cues signaling reward between healthy weight and obese adolescents. In this previous study, it was proposed that the SOT could additionally be used to index participants’ effort to acquire reward (Jonker et al., 2016). Although effort to acquire reward was not related to a concurrent high BMI, higher effort to acquire reward at age 16 was related to an increase in BMI between the age of 16 and 19 (Jonker et al., 2016). It might thus be that (heightened) effort to acquire reward does play a role in obesity. In the present study, we therefore complemented the self-report and the attention measures of reward sensitivity with this effort measure that can be extracted from participants’ performance on the SOT.

It has been suggested that overeating might also be related to individuals’ tendency to respond to reward while disregarding the negative consequences of their behavior (Danner et al., 2012). Consequently, not only reward sensitivity but also punishment sensitivity might play a role in obesity. Accordingly, obese adults have been found to report less sensitivity to punishment on the BIS/BAS than adults with a healthy weight (Danner et al., 2012). However, other studies reported no differences in punishment sensitivity as indexed by the BIS/BAS between obese and healthy weight adults (Schienle et al., 2009), and children (Nederkoorn et al., 2006), and no relationship between punishment sensitivity and BMI in adolescents (Jonker et al., 2016). Further, no relationship between attention for cues signaling punishment as indexed by the SOT and BMI was found (Jonker et al., 2016). Yet, it was found that participants’ performance on the SOT in terms of effort to prevent punishment was related to BMI (Jonker et al., 2016). Specifically, higher BMI was related to less effort to avoid punishment (i.e., relatively slow responses when they could avoid punishment), and this relatively low effort to avoid punishment was also related to an increase in BMI between the age of 13 and 19 (Jonker et al., 2016). All in all, there is some indication that next to high reward sensitivity also low punishment sensitivity might play a role in obesity. However, as with reward sensitivity this seems dependent on the component of punishment sensitivity that is studied. Since attention for cues signaling punishment, and effort to prevent punishment have so far only been examined in a population mainly consisting of healthy weight and overweight individuals, the current study will examine differences between healthy weight and obese adolescents on these measures. Further, for completeness, self-reported punishment sensitivity will also be assessed with the BIS/BAS.

In short, this study was designed to examine the role of reward and punishment sensitivity in obesity. Therefore, we compared healthy weight adolescents [adjusted BMI (actual BMI/Percentile 50 of BMI for age and gender) × 100) between 85 and 120%] to obese adolescents starting treatment (adjusted BMI > 140%). By specifically including treatment seeking adolescents with obesity and severe obesity we include a group that differs relatively extremely in BMI from healthy weight individuals. Consequently, if reward and punishment sensitivity play a clinically relevant role in adolescent obesity a difference should be evident between this group and a comparison group with a healthy weight. Further, to fully unravel the role of reward and punishment sensitivity, self-reported reward responsivity, reward drive, and punishment sensitivity, as well as behavioral measures of participants’ proneness to detect cues signaling reward/punishment, and of participants’ effort to obtain reward and avoid punishment were included in this study.

## Materials and Methods

### Participants

Participants were 51 adolescents with obesity (41 female, Mean age = 16.45, SD age = 1.63), and a comparison group consisting of 51 adolescents with a healthy weight (40 female, Mean age = 16.45, SD age = 1.87). Adolescents with obesity (i.e., adjusted BMI > 140%) between the ages of 12 and 23, who were referred for outpatient treatment to the eating disorder clinic of Accare in the Netherlands between June 2015 and June 2017 were eligible to participate. Additionally, adolescents between the ages of 15 and 18 who were referred for inpatient treatment to the treatment center Zeepreventorium De Haan in Belgium in 2016 were eligible to participate in this study. Of the 51 included patients with obesity, 19 were included in the Netherlands and 32 in Belgium. There was no difference in age [*t*(49) = −0.45, *p* = 0.652, Cohen’s *d* = 0.13], adjusted BMI [*t*(46) = 0.18, *p* = 0.861, Cohen’s *d* = 0.05], or educational level (χ*^2^* = 0.81, *p* = 0.369, *φ* = 0.13) between the group of patients that was recruited in the Netherlands and the group that was recruited in Belgium. The comparison group was matched on country, age, and gender to the adolescents with obesity. Due to a mix up during recruitment, one obese female was matched with a healthy weight male.

### Materials

#### Body Mass Index

Adjusted BMI was calculated [(actual BMI/Percentile 50 of BMI for age and gender) × 100]. The 50th percentile of BMI for age and gender was obtained from the Netherlands Organization for Applied Scientific Research (TNO, 2010). Adjusted BMI scores between 85% and 120% are considered as healthy weight, between 120 and 140% as overweight, and larger than 140% as obese; whereby >180% represents severe obesity, comparable with BMI of >40 in adults (Van Winckel and Van Mil, 2001).

#### Eating Disorder Symptoms

A Dutch translation of the 6th version of the Eating Disorder Examination Questionnaire (EDE-Q) (Fairburn and Beglin, 2008), was administered to assess eating disorder pathology within the past 28 days. Adaptations were made by the authors, comparable to adaptations that were made to the previous version of the EDE (Bryant-Waugh et al., 1996) to make it appropriate for children and adolescents. An average score of the 22 items of this questionnaire will be used as general index of eating disorder pathology (Aardoom et al., 2012), with higher scores reflecting more eating pathology. Internal consistency of this total EDE-Q score in the current study was excellent (Cronbach’s alpha of 0.95).

#### Self-Reported Reward and Punishment Sensitivity

Self-reported reward and punishment sensitivity was measured with the Behavioral Inhibition Scale/Behavioral Activation Scale (BIS/BAS; Carver and White, 1994). The BIS/BAS consists of 24 statements that can be answered on a 4-point scale ranging from *very false for me* (1) to *very true for me* (4). The questionnaire contains 4 filler items that were not used to index reward and punishment sensitivity. Further, there are 7 items on punishment sensitivity (BIS; e.g., “I worry about making mistakes”), 5 items on reward responsivity (BAS-RR; e.g., “When good things happen to me, it affects me strongly”), 4 items regarding reward drive (BAS-Dr; e.g., “I go out of my way to get things I want”), and 4 items regarding fun seeking (BAS-FS; e.g., “I crave excitement and new sensations”). As explained in the introduction, the BAS-FS is not of interest for the current study, but will be reported in the descriptives for the sake of completeness. Subscale scores are calculated by averaging the respective item scores. Internal consistency of the BIS, BAS-RR, and BAS-Dr were good (Cronbach’s alpha of 0.76, 0.70, 0.79 respectively), and of the BAS-FS poor (Cronbach’s alpha of 0.59).

#### Performance-Based Measures of Reward and Punishment Sensitivity

**FIGURE 1:**
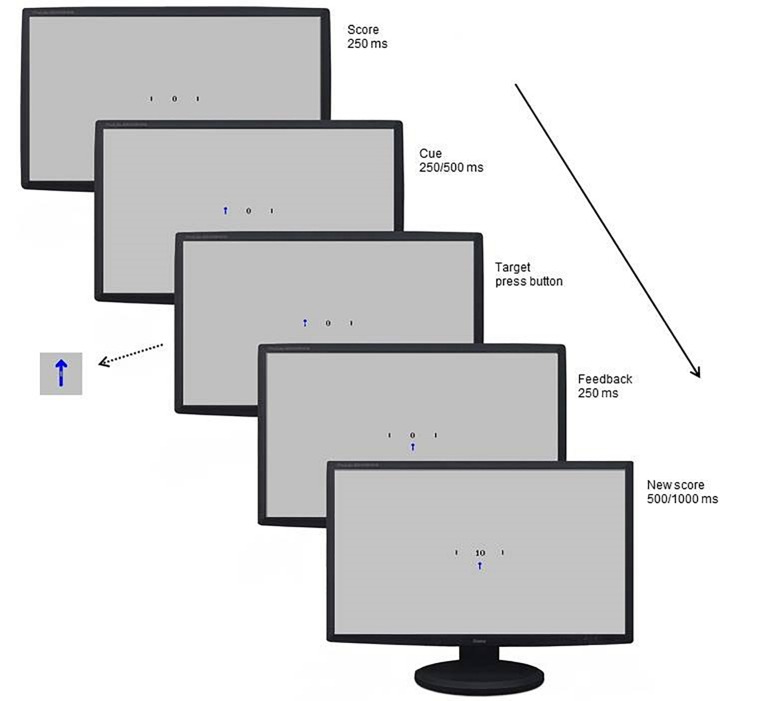
Example of a blue cue, cued trial with a sufficiently fast response in a winning game.

Attentional bias to reward and punishment was measured with the Spatial Orientation Task (SOT; Derryberry and Reed, 2002). The SOT is a reaction time task that indexes individuals’ tendency to direct their attention toward cues signaling reward and punishment (i.e., attentional engagement), and their difficulty to look away from cues signaling reward and punishment (i.e., attentional disengagement). Additionally, it differentiates between a more automatic attentional process that happens in a short time period (cue delay 250 ms), and a more voluntary process that happens over a somewhat longer time period (cue delay 500 ms). The SOT was completed on a HP Probook 650 G1 running Windows 7 on a 15-inch monitor (1366 × 768 pixels). Screen refresh rate was set at 60 Hz, and the task was programmed in E-prime 2.0 (Schneider et al., 2002). Participants were seated 50 cm away from the screen and responses were collected with a USB response box. Figure 1 shows an example of an SOT trial.

Throughout the task, individuals’ current score was shown in the middle of the screen, and participants were instructed to pay attention to this score during the game. Two black bars were displayed throughout the task, one on the right and one on the left side of this score. The start of a trial was indicated by the current score disappearing from the screen for 200 ms, and 250 ms after it reappeared, a cue replaced one of the two black bars. This cue was either a blue arrow pointing upward or a red arrow pointing downward. After 250 ms (short cue delay; i.e., more automatic process) or 500 ms (long cue delay; i.e., more voluntary process) a target (i.e., gray rectangle) appeared either in the arrow (cued trials) or in the remaining black bar (uncued trials). Blocks consisted of an equal amount of short and long cue delay time trials which were presented in random order. Participants were instructed to respond with a button press on the response box as soon as they saw the target. Two thirds of the targets appeared in the cued location, and one third in the uncued location. The blue cue signaled that responding to the cued target would be easy and it results in a fast enough response 75% of the time (see Table 1). Responding to the uncued target in a blue cue trial would be hard and results in an insufficiently fast response 75% of the time. For the red cue it is the opposite, responding to the cued target would be hard and it results in an insufficiently fast response 75% of the time. Responding to an uncued target in a red cue trial would be easy and results in a fast enough response 75% of the time. Thus, in general the blue cue was a signal for a high chance of a fast enough response, and the red cue a signal for a high chance of a too slow response. Participants were informed about this difference between the cues in the instructions. In some trials no target appeared (i.e., catch trials), and for those trials, participants were instructed to not press the button. The task consisted of winning and losing blocks. During winning blocks, participants could win 10 points for every sufficiently fast response, and their score remained unchanged after an insufficiently fast response. During losing blocks, insufficiently fast responses resulted in the loss of 10 points, and sufficiently fast responses did not change participants score. In both types of blocks, participants lost 10 points for responding on catch trials or responding before the target appeared.

**TABLE 1:** Overview of trials of the spatial orientation task.

**Cue**	**Target**	**Odds**	**Cue delay time**	**Cutoff for fast response^a^**	**Correction for cue delay time**	**Anticipated outcome**
Blue	Cued	2/3	250 ms	Median RT **+** 0.55 SD	+12 ms	75%chance of **positive** outcome
	Cued	2/3	500 ms	Median RT **+** 0.55 SD	–12 ms	75% chance of **positive** outcome
	Uncued	1/3	250 ms	Median RT – 0.55 SD	+12 ms	75%chance of **negative** outcome
	Uncued	1/3	500 ms	Median RT – 0.55 SD	–12 ms	75% chance of **negative** outcome
Red	Cued	2/3	250 ms	Median RT – 0.55 SD	+12 ms	75%chance of **negative** outcome
	Cued	2/3	500 ms	Median RT – 0.55 SD	–12 ms	75% chance of **negative** outcome
	Uncued	1/3	250 ms	Median RT **+** 0.55 SD	+12 ms	75%chance of **positive** outcome
	Uncued	1/3	500 ms	Median RT **+** 0.55 SD	–12 ms	75% chance of **positive** outcome

**RT, reaction time. ^*a*^Since the cutoff score is calculated relative to performance, this is not expected to influence performance of some individuals differently than performance of others.**

Participants were presented with a feedback signal at the end of each trial, again using the blue and red arrow. The blue arrow pointing upwards signaled a sufficiently fast response on targeted trials or a correct non-response on catch trials. The red arrow pointing downward signaled an insufficiently fast response on targeted trials or an inappropriate response on catch trials. This feedback signal was shown below the score. For the practice blocks, a fixed cutoff of 350 ms was used to identify sufficiently fast responses. In the blocks following, personalized cutoff scores were used to indicate sufficiently fast responses. That is, the median reaction time and standard deviation of the previous block of the same type was used to calculate the cutoff. During cued blue or uncued red trials, responses were labeled sufficiently fast when they were faster than participant’s median reaction time plus 0.55 times the standard deviation. During uncued blue or cued red trials, responses were labeled sufficiently fast when they were faster than participant’s median reaction time minus 0.55 times the standard deviation. Further, 12 ms were added to the median reaction time for short-delay trials and 12 ms were subtracted from the median reaction time for long-delay trials to control for average differences between short and long cue delay trials (Derryberry and Reed, 2002; see Table 1 for an overview).

The task started with an instruction block, with 7 cued, 6 uncued, and 1 catch trial, all trials with a long delay after the cue. This instruction block was followed by two practice blocks – a winning and a losing – each consisting of 6 cued, 6 uncued, and 2 catch trials. After the practice blocks, all participants started the test with two winning games, continued with two losing games followed by another two winning and two losing games. Each game consisted of 32 cued trials (57%), 16 uncued trials (29%), and 8 catch trials (14%) in random order.

In order to emphasize reward during the winning games, participants were told that they could win a prize (i.e., reward) if they performed well on the games where they could win points. Additionally, to emphasize punishment during the losing games, participants were told they would have to redo the task (i.e., punishment) if they did not perform well enough on the games where they could lose points. In order to give the impression that this was checked by the researcher, participants had to write their obtained block scores on a score form. At the end, all participants won a prize (i.e., gift bag with a mug, notebook and pencil for girls, and a mug with a funny text for guys), and were told that they performed well enough to not have to redo the task.

Following the task, but after they were informed that they won the prize and did not have to redo the task, participants answered six questions to examine assumptions made in the tasks design. To examine whether the reward (i.e., winning a prize), and the punishment (i.e., redoing the task) were comparable in strength, participants were asked how much they liked that they could win a prize, and how much they disliked that they might had to redo the task. These two questions were answered on a VAS ranging from *Not at all* (0) to *A lot* (100). To examine whether the blue cue became a signal of reward, and the red cue a signal of punishment, participants were asked how they felt about the blue and the red arrow. These two questions were answered on a VAS ranging from *Negative* (0) to *Positive* (100). To examine whether the blue trials were experienced as more easy than the red trials, they were asked whether it was easy to respond fast enough in blue arrow trials, and red arrow trials. These two questions were answered on a VAS ranging from *Completely disagree* (0) to *Completely agree* (100).

The SOT data was reduced following Jonker et al. (2016). That is, in this study we inferred reward sensitivity from the winning games and punishment sensitivity from the losing games since reward was emphasized in the winning games and punishment in the losing games. Attentional engagement to reward was inferred when, during winning games, participants responded faster to targets that appeared in the location of the blue cue (signaling reward) than to targets that appeared in the location of the red cue (signaling non-reward). Higher scores reflect more attentional engagement with reward. Difficulty to disengage from reward was inferred when participants, during winning games, responded slower in uncued trials with a blue cue than in uncued trials with a red cue. Higher scores reflect more difficulty to disengage from reward. Attentional engagement to punishment was inferred when, during losing games, participants responded faster to targets that appeared in the location of the red cue (signaling punishment) than to targets that appeared in the location of the blue cue (signaling non-punishment). Higher scores reflect more attentional engagement with punishment. Difficulty to disengage from punishment was inferred when, during losing games, participants responded slower on uncued red trials than on uncued blue trials. Higher scores reflect more difficulty to disengage from punishment (see Appendix Table A1).^1^

Furthermore, effort was indexed by taking the overall speed of responses on the winning games and on the losing games. Overall speed was calculated by averaging reaction times of all trials (cued, uncued, short cue delay time, and long cue delay time) of the winning and losing games separately. Higher scores, meaning slower RTs, on effort mean relatively low effort to obtain reward, and relatively low effort to obtain punishment. Since the speed of the responses might change over the course of the game, for example due to a learning effect or decreased motivation, effort scores were calculated separately for the first half and the second half of the game (cf., Jonker et al., 2016).

##### Data reduction

Before calculating the attentional bias measures of the SOT, outliers and errors were removed, following Van Hemel-Ruiter et al. (2013). First, trials on which participants responded before the target appeared were deleted. For the obese group this resulted in the deletion of 8.0% of the trials, and for the healthy weight group this was 7.8%. Trials during which participants did not respond whereas they should have responded (omission errors) were also excluded from further analyses. This were 10.2% of the trials in the obese adolescents group and 6.3% of the trials in the healthy weight adolescents group. Trials with responses after the target appeared but with reaction times below 125 ms (anticipation errors) were deleted. In the obese adolescents group this applied to 8.1% of the trials, and in the healthy weight adolescents group 7.5%. No trials with reaction times above 1000 ms (probable distractions) were identified. Mean reaction times and standard devations after this data reduction procedure can be found in Appendix Table A2.

Strikingly, after deletion of these outliers and errors, there were four participants that had missing data points. This means that for these participants not one trial of the 16 (uncued) or 32 (cued) was left for some of the trial types (e.g., winning games, cued blue, short cue delay trials). Because of this unexpected finding, we examined the data more closely and found that for several other participants – for some types of trials – only very few trials were left as well. Therefore, we decided to perform an additional analysis for the subsample that had a sufficient number of correct responses (i.e., errors on less than half of the trials of each trial type) on top of the planned analyses. This subsample consists of 35 healthy weight and 28 obese individuals. The group with a high error rate did not differ from the group with a lower error rate in age [*t*(94) = −0.39, *p* = 0.700, Cohen’s *d* = 0.08], or educational level (χ*^2^* = 1.89, *p* = 0.170, φ = 0.14). There was also no difference in BMI between the individuals with high and low error rates in the healthy weight group [*t*(46) = 0.88, *p* = 0.382, Cohen’s *d* = 0.24], or in the obese group [*t*(46) = −0.17, *p* = 0.863, Cohen’s *d* = 0.05].

##### SOT response pattern

Paired samples *t*-tests showed faster responses on cued blue trials than on cued red trials suggesting a general engagement effect for cues signaling reward. The speed of responses did not differ between uncued blue and uncued red trials, providing no evidence for a disengagement effect in on any of the trial types (Table 2). The same pattern was found in the subgroup analyses (see Appendix Table A3).

**TABLE 2:** Overall differences between blue and red cue trials, separately for different trial types.

				**95% confidence interval of the Difference**		
		**Calculation**	**Cue delay**	**Lower bound**	**Upper bound**	***p***
WG	Attentional engagement	Cued red – cued blue	Short	30.41	46.50	<0.001
			Long	20.77	44.42	<0.001
	Attentional disengagement	Uncued blue – uncued red	Short	–28.89	3.21	0.116
			Long	–12.47	13.47	0.939
LG	Attentional engagement	Cued blue – cued red	Short	–41.17	–24.48	<0.001
			Long	–44.46	–19.52	<0.001
	Attentional disengagement	Uncued red – uncued blue	Short	–7.87	26.11	0.289
			Long	–22.96	8.62	0.370

**N = 96. WG, Winning Game; LG, Losing Game.**

##### SOT task assumptions

Mean scores on the task assumption questions are shown in Table 3. In general, participants were more positive about the possibility of winning a prize, than they were negative about the possibility that they might have to redo the task [*t*(95) = 3.99, *p* < 0.001, Cohen’s *d* = 0.52]. Further, the blue arrow was rated as more positive than the red arrow [*t*(95) = 16.28, *p* < 0.001, Cohen’s *d* = 2.80], and blue cue trials were rated as more easy than red cue trials [*t*(95) = 9.69, *p* < 0.001, Cohen’s *d* = 1.48]. Since the evaluation of these task aspects might be influenced by individuals’ reward and punishment sensitivity, and we expected group differences on sensitivity to reward and punishment, it was examined whether the obese and healthy weight adolescents differed in their answers on these questions. Interestingly, the obese individuals were more negative about the punishment they could receive than the healthy weight individuals, but the groups did not differ in how positive they were about the option of winning a prize. Furthermore, obese individuals rated the red arrow as less negative than the healthy weight individuals. There was no difference between the groups in how they rated the blue arrow, or how easy they thought it was to respond to the blue and red cue. Thus with regard to the reward aspects of the task there were no differences between the groups. However, it seems that the overall punishment of having to redo the task was perceived as more punishing to obese participants, but the cue signaling punishment was perceived as less punishing to obese participants.

**TABLE 3:** Checking task assumption questions.

	**All (*N* = 96)**	**Healthy weight (*N* = 48)**	**Obese (*N* = 48)**	**Between-groups test**
	
	***Mean* (*SD*)**	***Mean* (*SD*)**	***Mean* (*SD*)**	***t* (*p*)**
How much did you like that you could win a prize	79 (20)	81 (18)	78 (23)	0.60 (0.550)
How much did you mind that you might had to redo the task	65 (34)	58 (29)	72 (36)	−2.21 (0.030)
I think the blue arrow was	77 (20)	77 (18)	76 (22)	0.36 (0.718)
I think the red arrow was	21 (20)	16 (15)	26 (23)	−2.39 (0.019)
It was easy to respond in blue cue trials	67 (25)	70 (23)	64 (26)	1.28 (0.202)
It was easy to respond to red cue trials	29 (27)	28 (25)	30 (28)	−0.28 (0.781)

### Procedure

The part of the study that was performed in the Netherlands was approved by the Medical Ethical Committee of the University Medical Center in Groningen, the Netherlands (NL.51694042.14). The part that was performed in Belgium was approved by Ghent University’s Ethics Committee (2015/88). Participants, and their parents when they were under 18 years of age, signed informed consent. For patients, the study took place at the treatment center and for the comparison group the study took place at their school, at Ghent University, or at their own home in a quiet room. For all participants testing was supervised by a trained researcher. Participants performed the SOT after which they answered the EDE-Q and the BIS/BAS. Two obese participants did not answer the questionnaires due to a time constraint, and the SOT task of one obese and one healthy weight participant crashed and these data are therefore missing. After finishing the questionnaires, participants’ height and weight were measured. Height and weight of obese patients recruited in Belgium were taken from their patient file, reporting height and weight assessment a maximum of 1 week before the study.

This study describes data from a larger project on characteristics that might play a role in disordered eating behavior. Participants performed several reaction time measures of which the SOT was the fourth and last. The procedure for the obese and healthy weight adolescents was identical.

### Analyses

Group differences between adolescents with obesity and the comparison group on age, adjusted BMI, and EDE-Q score were assessed with independent samples *t*-tests. Differences in educational level was assessed with the Chi-square test.

To examine whether adolescents with obesity differ in sensitivity to reward from adolescents with a healthy weight (1) a Multivariate Analysis of Variance (MANOVA) was performed with BAS-RR and BAS-Drive as dependent variables and Group (obese or healthy weight) as fixed factor, (2) a MANOVA was performed with the four attentional bias scores – engagement to cues signaling reward on the short and long cue delay and disengagement from cues signaling reward on the short and long cue delay – as dependent variables and Group (obese or healthy weight) as fixed factor. If a MANOVA showed a significant overall effect, univariate ANOVAs were used to examine on which variable(s) differences were found between the groups. To correct for familywise error rate a Bonferroni-Holm correction was applied in these cases.

To examine whether adolescents with obesity are less sensitive for punishment than the healthy weight group (1) an ANOVA was performed to compare BIS scores of healthy weight and obese adolescents, (2) a MANOVA was performed with the four attentional bias scores – engagement to cues signaling punishment on the short and long cue delay and disengagement from cues signaling punishment on the short and long cue delay –as dependent variables, and Group (obese or healthy weight) as fixed factor. Univariate ANOVAs were used to follow-up on significant overall effects on the MANOVA. To correct for familywise error rate a Bonferroni-Holm correction was applied in these cases.

Lastly, to examine whether adolescents with obesity differ from individuals with a healthy weight on the relative effort they put into the performance during rewarding games and the performance during punishing games, a Repeated Measures Analysis of Variance (RM-ANOVA) was performed with Game type (losing vs. winning) and Timing (first half vs. second half) as within subject factors and, and Group (obese or healthy weight) as between subjects factor.

The ANOVA’s and the RM-ANOVA had a power of 70% to find medium effects (G^∗^Power; Faul et al., 2007). The sample thus provides us with sufficient power to find practically meaningful effects. To increase the confidence in our results and test the evidence for the null-hypotheses in the case of non-significant findings, classical statistical analyses were complemented with results following the Bayesian approach. Bayesian analyses were conducted with JASP (JASP Team, 2018). Only *t*-tests were performed, since there is no option for a Bayesian MANOVA. Cauchy prior was set at the recommended default *r* = 0.707 (Wagenmakers et al., 2017). To facilitate interpretation of the outcomes, BF_10__,_ which quantifies the evidence for the alternative hypotheses over the null hypotheses (e.g., adolescents with obesity differ in sensitivity to reward from adolescents with a healthy weight), were reported. A Bayes factor of 1 is considered *no evidence*, between 1 and 3 *anecdotal*, between 3 and 10 *moderate*, between 10 and 30 *strong*, between 30 and 100 *very strong*, and more than 100 *extreme* evidence that the data are more likely under the alternative hypothesis. A Bayes factor between 1/3 and 1 *anecdotal*, 1/10 and 1/3 *moderate*, 1/30 and 1/10 *strong*, 1/100 and 1/30 *very strong*, and less than 1/100 *extreme* evidence that the data are more likely under the null hypothesis (Wagenmakers et al., 2017).

## Results

### Group Characteristics

Table 4 shows educational level, and the mean age, adjusted BMI and EDE-Q scores of the healthy weight and obese adolescents. Adjusted BMI of the adolescents with obesity ranged between 143 and 313%, and of the adolescents in the comparison group between 75 and 129%. The difference in adjusted BMI between the two groups was large (Cohen’s *d* = 3.18), and adolescents with obesity scored higher on eating disorder symptoms than the healthy weight adolescents (Cohen’s *d* = 1.25). No difference was found between the age of the two groups, which is in line with the individual matching procedure. The chi square test showed a weak but significant difference in educational level, meaning that the proportion of adolescents with lower educational level was higher in the obese group compared with the healthy weight comparison group (φ = −0.21).

**TABLE 4:** Group characteristics.

	**Healthy weight (*n* = 51)**		**Obese (*n* = 51)**		**Between-groups test**
Educational level^a^	Low	23	Low	34	*X ^2^* = 4.81, *p* = 0.046
	High	28	High	17	
	
	**Mean**	**SD**	**Mean**	**SD**	***t* (*p*)**
	
Age	16.45	1.87	16.45	1.63	0.00 (1.00)
BMI	101.79	9.95	180.63	33.61	16.06 (<0.001)
EDE-Q	1.17	1.35	2.70	1.09	6.24 (<0.001)

**BMI, Adjusted Body Mass Index; EDE-Q, Total score on the Eating Disorder Examination Questionnaire. ^*a*^Since the International Standard Classification of Education highly depends on the amount of years of education an individual has had, this classification does not seem appropriate in a sample with such a large age range as the current sample. Therefore educational level was indexed as either low (i.e., education preparing for or on the level of practically-based, occupationally-specific and preparing for labor market entry) or high (i.e., education preparing for or on the level of bachelor or master level qualification).**

### Descriptives

Table 5 shows the mean scores on the self-report measures, attentional bias scores and effort scores of the adolescents with a healthy weight and adolescents with obesity. Since adolescents with obesity and healthy weight adolescents differed in their educational level we examined the relationship between educational level and outcome measures. However, educational level was not related to the self-reported reward or punishment sensitivity (−0.14 < *r_s_* > 0.04; 0.153 < *p* > 0.917), the attentional bias scores (−0.16 < *r_s_* > 0.10; 0.127 < *p* > 1.000), or the effort indexes (−0.13 < *r_s_* > 0.06; 0.218 < *p* > 0.590). Correlations between all continuous variables can be found in Appendix Table A4.

**TABLE 5:** Mean scores of reward and punishment sensitivity per group.

	**Healthy weight (*n* = 51)**	**Obese (*n* = 49)**	**Between-groups test**
	
	**Mean (*SD*)**	**Mean (*SD*)**	***BF*_10_**
**Self-Report**			
BAS-RR	3.30 (0.42)	3.09 (0.54)	1.92
BAS-Drive	2.66 (0.67)	2.63 (0.69)	0.22
BAS-FS	2.95 (0.50)	2.86 (0.63)	–
BIS	2.90 (0.56)	2.85 (0.62)	0.23

	**Healthy weight (*n* = 48)**	**Obese (*n* = 48)**	

**Attentional Bias**			
Reward engagement 250 ms	37.22 (36.03)	36.70 (43.41)	0.21
Reward engagement 500 ms	35.40 (50.80)	29.79 (65.47)	0.25
Reward disengagement 250 ms	−9.99(69.93)	−15.69(88.17)	0.23
Reward disengagement 500 ms	−0.88(54.65)	1.88 (72.76)	0.22
Punishment engagement 250 ms	−31.35(38.89)	−34.30(43.72)	0.21
Punishment engagement 500 ms	−27.09(53.62)	−36.99(69.22)	0.23
Punishment disengagement 250 ms	−6.37(70.07)	24.62 (93.86)	0.21
Punishment disengagement 500 ms	−6.55(71.91)	−7.79(84.30)	0.91
**Effort**			
Reward first half	386.44 (51.52)	411.06 (52.91)	–
Reward second half	366.41 (55.41)	402.15 (46.29)	–
Punishment first half	379.40 (49.68)	406.85 (51.17)	–
Punishment second half	359.61 (51.29)	391.81 (53.49)	–

**BIS, punishment sensitivity of the BIS/BAS; BAS-RR, Reward responsivity of the BIS/BAS; BAS-Dr, Reward drive of the BIS/BAS; BAS-FS, Fun Seeking of the BIS/BAS; –, not, or not directly, tested.**

### Do Obese Adolescents Differ From Healthy Weight Adolescents in Reward Sensitivity?^2^

#### Self-Report

A significant difference was found between adolescents with obesity and adolescents with a healthy weight on self-reported reward sensitivity [*F*(2, 97) = 3.19, *p* = 0.046, η*^2^_p_* = 0.06]. Between subjects test showed that adolescents with obesity scored lower on reward responsivity [*F*(1, 98) = 5.03, *p* = 0.027, η*^2^_p_* = 0.05], but not reward drive [*F*(1, 98) = 0.05, *p* = 0.830, η*^2^_p_* = 0.00], than adolescents with a healthy weight. However, after applying the Bonferroni-Holm correction, and thus testing against α = 0.025, this difference was only marginally significant. Further, the Bayes factor shows that there is only anecdotal evidence for a difference in reward responsivity between obese and healthy weight adolescents (Table 5).

#### Attentional Bias

The groups did not differ on attentional bias to cues signaling reward [*F*(4, 91) = 0.15, *p* = 0.962, η*^2^_p_* = 0.01]. The Bayes factors show that there is moderate evidence that the data are more likely under the null hypothesis (Table 5). These results were similar when only the subgroup of participants with a sufficient amount of correct trials were compared [*F*(4, 55) = 0.96, *p* = 0.962, η*^2^_p_* = 0.01].

### Are Obese Adolescents Less Sensitive to Punishment Than Healthy Weight Adolescents?^2^

#### Self-Report

The independent samples *t*-test did not show a significant difference between healthy weight and obese adolescents on BIS [*F*(1, 98) = 0.23, *p* = 0.634].

#### Attentional Bias

There was no significant difference between the groups on attention for cues signaling punishment [*F*(4, 91) = 0.88, *p* = 0.481, η*^2^_p_* = 0.04]. The Bayes factor shows that there is moderate evidence that the observed data on engagement to cues signaling punishment on the short and long cue delay trials, and disengagement from cues signaling punishment on the long cue delay trials are more likely under the null hypothesis, and anecdotal evidence that the observed data on disengagement from cues signaling punishment on the short delay trials are more likely under the null hypothesis. Similar outcomes were observed when only the participants with a sufficient amount of correct trials were compared [*F*(4, 55) = 0.32, *p* = 0.864, η^2^*_p_* = 0.02].

### Do Adolescents With Obesity Differ in the Effort They Put Into Obtaining Reward vs. Avoiding Punishment From Adolescents With a Healthy Weight?^2^

Participants became faster on the SOT trials over the course of the game as shown by a main effect of Time [*F*(1, 94) = 20.65, *p* < 0.001, η^2^*_p_* = 0.18, *BF*_10_ = 270,473], and they were slower on winning than on losing games as revealed by a main effect of Game type [*F*(1, 94) = 8.55, *p* = 0.004, η^2^*_p_* = 0.09, *BF*_10_ = 1.84]. There was no difference between obese and healthy weight adolescents in the change in speed over time as shown by a non-significant interaction effect of Time × Group [*F*(1, 94) = 1.28, *p* = 0.261, η^2^*_p_* = 0.01, *BF*_10_ = 0.39]. Obese adolescents were slower on both winning and losing games, than healthy weight adolescents as shown by the significant main effect of Group [*F*(1, 94) = 10.33, *p* = 0.002, η^2^*_p_* = 0.10, *BF*_10_ = 18.53]. There was no significant interaction between Game Type × Group, showing that this difference was similar for the winning and losing games [*F*(1, 94) = 0.01, *p* = 0.942, η^2^*_p_* < 0.001, *BF*_10_ = 0.15].

## Discussion

This study was set out to examine the role of reward and punishment sensitivity in obesity among adolescents. We complemented self-report measures with indices of attentional bias for cues signaling reward and punishment, and measures of effort to obtain reward and avoid punishment. Findings can be summarized as follows: (1) obese adolescents did not significantly differ in reward responsivity, reward drive or attention for cues signaling reward; (2) obese adolescents did not report less punishment sensitivity than healthy weight adolescents, nor did they show less attention bias for cues signaling punishment; and, (3) obese adolescents showed less effort to obtain reward and less effort to avoid punishment than adolescents with a healthy weight.

Adolescents with obesity had a tendency to report less responsivity to reward than adolescents with a healthy weight, which seems to be in line with the RDS theory that posits that obese individuals might overeat as the result of experiencing less feelings of reward (i.e., low reward responsivity). However, after correction for family wise error rate the difference was only marginally significant, and the outcomes of the Bayesian analyses showed that the strength of the evidence for this finding should be considered inconclusive. Consequently, there does not seem to be clear evidence that obese adolescents differ in reward responsivity from adolescents with a healthy weight. These findings seem consistent with a study reporting on the linear relationship between reward responsivity and BMI in a large sample of adolescents, where no relationship between reward responsivity and BMI was found, and reward responsivity was not related to increases in BMI between the age of 13 and 19 (Jonker et al., 2016). Perhaps in that large study no relationship was found because only a small proportion of obese adolescents was included in that study (2.7%). However, taken together with the outcomes of the current study, in which we included a substantial group of obese and severely obese adolescents, the findings point to the conclusion that obese adolescents do not differ in general reward responsivity from healthy weight adolescents. This conclusion also seems consistent with an fMRI study that showed that aberrant reward region responsivity did not predict future weight gain (Stice et al., 2013). Future studies might focus on responsivity to more specific cues that are relevant to the behavior of obese adolescents. That is, even though a general responsivity might be unrelated to obesity, responsivity to food might be related. This view is consistent with a previously found inverse relationship between BMI and brain activation in response to food reward (Stice et al., 2008).

Adolescents with obesity did not differ from healthy controls in their reported drive to obtain reward. These findings are in line with a previous study showing no relationship between reward drive and BMI (Jonker et al., 2016), but in apparent contrast with a study reporting a positive relationship (De Decker et al., 2016), and a study reporting a quadratic relationship (Verbeken et al., 2012). Importantly, only the last study included a substantial amount of obese individuals. The quadratic relationship as reported by Verbeken et al. (2012), showed a positive relationship between drive and BMI up to the average adjusted BMI of overweight adolescents (adjusted BMI of 133), after which a negative relationship was reported. Consequently, the results of Verbeken et al. (2012) can still be consistent with the positive relationship reported previously by De Decker et al. (2016) in healthy weight and overweight adolescents, and the finding of the current study that obese adolescents did not differ in reward drive from healthy weight adolescents. Nevertheless, although heightened reward drive might be related to overeating and the development of overweight, the findings of the current study add to the evidence that adolescents with obesity do not seem to have heightened reward drive (anymore).

Our results showed no difference between adolescents with obesity and adolescents with a healthy weight in self-reported punishment sensitivity. That is, obese adolescents do not seem to be more or less sensitive to punishment than adolescents with a healthy weight. This is in line with prior findings in children (Nederkoorn et al., 2006), but in contrast with a study in which obese adults were found to report lowered sensitivity to punishment than adults with a healthy weight (Danner et al., 2012). This apparent inconsistency might reflect differences in age between these samples. More specifically, it might be that the development of obesity in these different age groups is the result of different characteristics and that whereas adult obesity is characterized by lowered punishment sensitivity, punishment sensitivity does not play a role in childhood and adolescent obesity. However, it is also possible that lowered punishment sensitivity as seen in adults is a consequence of being obese and as such this is not found in adolescents who are likely suffering from obesity for a shorter period of time. Another explanation for the apparent inconsistencies might be differences in severity of eating disorder symptoms within the samples of obese patients in these studies. The obese sample in the current study reported significantly more severe/frequent symptoms of eating disorders than the healthy weight comparison group. Previously, a positive relationship has been reported between eating disorder symptoms and punishment sensitivity (Matton et al., 2013), and *post hoc* correlational analyses also showed a positive relationship between punishment sensitivity and eating disorder symptoms in the current obese sample (*r* = 0.39, *p* < 0.01). Thus, even when overeating would be related to lowered punishment sensitivity, the moderate positive relationship between punishment sensitivity and eating disorder symptoms might have clouded this relationship. Since previous studies did not report on eating disorder symptoms of their samples (Nederkoorn et al., 2006; Danner et al., 2012), it remains to be seen to what extent the findings in these studies were affected by eating disorder symptomatology. Future studies should further explore the interplay between severity/frequency of eating disorder symptoms, overeating, age and punishment sensitivity.

The current study showed no differences in attention for cues signaling reward and punishment between adolescents with a healthy weight and adolescents with obesity. A previous study similarly failed to find evidence for a relationship between BMI and attention for cues signaling reward and punishment (Jonker et al., 2016). Together these findings seem to converge to the conclusion that heightened attention for general cues signaling reward and punishment might not play a critical role in the development and maintenance of obesity.

The last aim of the study was to compare obese and healthy weight adolescents on their effort in terms of overall response time to prevent receiving punishment (i.e., losing points) and to obtain reward (i.e., winning points) during the SOT. The current finding that obese adolescents were slower to respond on games where they could receive a punishment than the healthy weight comparison group is in line with previous findings that lower effort to avoid punishment was related to a concurrent higher BMI (Jonker et al., 2016). However, the finding that obese adolescents were also slower on games were they could obtain reward seem in apparent contrast with previous findings that higher effort to obtain reward was related to an increase in BMI (Jonker et al., 2016). Perhaps, the most parsimonious interpretation of the effort findings is that obese adolescents were just slower in general, regardless of the content of the task. Such interpretation would be in line with a previous study in which obese individuals were overall slower than healthy weight individuals on an – albeit different – attentional task (Kemps et al., 2014). Further, it relates to previous findings showing that lower performance on gross motor coordination predicted an increase in BMI, and that weight status negatively influenced gross motor coordination (D’Hondt et al., 2014). It would help to examine the effort to obtain reward or avoid punishment relative to effort on neutral trials. However, since there is no neutral game in the SOT this is not possible with respect to the current data. Thus for future research it might be worthwhile to consider other behavioral tasks. A potential alternative option might be to use the Point-Scoring Reaction Time Task (Colder et al., 2011), which measures the influence of reward and punishment on how hard participants are working for the task at hand.

The current study has several strengths such as the matched comparison group and the comprehensive examination of reward and punishment sensitivity by using self-report as well as a performance measure of attention to cues that signal reward and punishment, and of effort to obtain reward and avoid punishment. However, there are also a couple of limitations that should be taken into account when interpreting the results. First, the sample is relatively small, providing a power of 70% to find medium sized effects. As a consequence of the current sample size, the current study had not sufficient power to reliably test potential gender differences. Since there is some indication that the role of reward and punishment sensitivity might be different for males and females, future studies should further explore potential gender differences (Dietrich et al., 2014). Further, a meta-analysis could be considered to overcome the issue of small sample size. However, if the aim is to understand the relationship between reward sensitivity and obesity, the low amount of obese individuals in the samples of several studies remain an issue. Second, the cross-sectional design of this study precludes the possibility to draw conclusions about the direction of the found relationships. Third, the SOT was subject to a high number of errors and outliers in the current sample. Since the task has previously been used in a similar age group this does not seem to be due to the age of the participants (Jonker et al., 2016), and might instead reflect participants’ motivation. Even so, findings did not seem to be different when excluding participants with many errors and outliers. Fourth, although a concrete reward and punishment were introduced during the SOT, the prospect of winning a prize or having to redo the task might be relatively weak in comparison to real-life rewards and punishments. Last, educational level of the two groups was not fully matched. Although there did not seem to be a relationship between educational level and reward and punishment sensitivity, we cannot rule out that this might have influenced our findings.

## Conclusion

To conclude, the current study was set out to investigate the role of reward and punishment sensitivity in obesity among adolescents. We complemented self-reported reward and punishment sensitivity with behavioral measures, and differentiated between reward responsivity and drive. Individuals with obesity did not seem to differ in reward responsivity, reward drive and attention for cues signaling reward from adolescents with a healthy weight. In addition, no difference was found between healthy weight and obese adolescents in self-reported punishment sensitivity or in attention for cues signaling punishment. Future studies should examine whether obese adolescents might be sensitive to reward from relevant stimuli such as food.

## Data Availability Statement

The datasets generated for this study are available on request to the corresponding author.

## Ethics Statement

The part of the study that was performed in Netherlands was approved by the Medical Ethical Committee of the University Medical Center in Groningen, Netherlands (NL.51694042.14). The part of the study that was performed in Belgium was approved by Ghent University’s Ethics Committee (2015/88). Participants, and their parents when they were under 18 years of age, signed informed consent.

## Author Contributions

NJ, KG, and PJ made substantial contributions to the conception and design of the study. NJ, EM, KG, LV, LG, and CB made substantial contributions to the acquisition of data for the work. NJ performed the statistical analysis and wrote the first draft of the manuscript. NJ, EM, KG, LV, LG, CB, and PJ contributed to manuscript revision, read and approved the submitted version, and agreed to be accountable for all aspects of the work in ensuring that questions related to the accuracy or integrity of any part of the work are appropriately investigated and resolved.

## Conflict of Interest

The authors declare that the research was conducted in the absence of any commercial or financial relationships that could be construed as a potential conflict of interest.

## Footnotes

1 This approach differs slightly, but only in interpretation, from the study of Van Hemel-Ruiter et al. (2013) in which sensitivity to cues signaling non-punishment was inferred from the losing games. That is, the losing games were also used as proxy to measure relative reward sensitivity. However, since in this study the cue signaling reward is the most emphasized cue in the winning games, and the cue signaling punishment is the most emphasized cue in the losing games we approach the losing games as measure of punishment.

2 Results of the correlational analyses as shown in Appendix Table A4 largely mirror these group comparisons. However, the correlational analyses with BMI as shown in Appendix Table A4 should be interpreted with caution since the assumptions of normality, linearity and homoscedasticity were violated.

## APPENDIX

**TABLE A1:** Calculation of attentional biases to reward and punishment.

**Game**	**Bias**	**Calculation**	**Interpretation**	**Cue delay time**	
Winning game	Attentional engagement	Mean RT cued red trials –mean RT cued blue trials	High score = high AB to reward	250 ms	Automatic
				500 ms	Voluntary
	Difficulty to disengage	Mean RT uncued blue trials – mean RT uncued red trials	High score = high AB to reward	250 ms	Automatic
				500 ms	Voluntary
Losing game	Attentional engagement	Mean RT cued blue trials – mean RT cued red trials	High score = high AB to punishment	250 ms	Automatic
				500 ms	Voluntary
	Difficulty to disengage	Mean RT uncued red trials – mean RT uncued blue trials	High score = high AB to punishment	250 ms	Automatic
				500 ms	Voluntary

**From Jonker et al. (2016). RT, reaction time; AB, attentional bias.**

**TABLE A2:** Mean reaction times and standard deviations of the Spatial Orientation Task.

	**NW (*n* = 48)**				**OB (*n* = 48)**			
	**Cued**		**Uncued**		**Cued**		**Uncued**	
	**Blue**	**Red**	**Blue**	**Red**	**Blue**	**Red**	**Blue**	**Red**
	**Short Cue Delay Time (250 ms)**							
WG	319 (44)	356 (47)	481 (83)	491 (90)	339 (49)	374 (45)	497 (101)	522 (79)
LG	316 (44)	347 (44)	477 (92)	471 (81)	341 (38)	381 (49)	515 (102)	530 (91)
	**Long Cue Delay Time (500 ms)**							
WG	334 (76)	369 (70)	394 (75)	395 (78)	357 (63)	394 (81)	441 (84)	433 (83)
LG	336 (69)	363 (75)	389 (79)	382 (71)	368 (77)	397 (80)	443 (97)	442 (80)

**WG, winning game; LG, losing game.**

**TABLE A3:** Overall differences between blue and red cue trials, separately for different trial types.

				**95% confidence interval of the difference**		
		**Calculation**	**Cue delay**	**Lower bound**	**Upper bound**	***p***
WG	Attentional engagement	Cued red – cued blue	Short	20.48	39.75	< 0.001
			Long	15.59	40.74	< 0.001
	Attentional disengagement	Uncued blue – uncued red	Short	–14.49	17.46	0.853
			Long	–9.64	19.43	0.503
LG	Attentional engagement	Cued blue – cued red	Short	–30.35	–13.44	< 0.001
			Long	–33.95	–8.52	< 0.01
	Attentional disengagement	Uncued red – uncued blue	Short	–22.62	10.97	0.491
			Long	–15.31	10.29	0.696

**N = 60. WG, Winning Game; LG, Losing Game.**

**TABLE A4:** Correlations between continuous variables.

	**1.**	**2.**	**3.**	**4.**	**5.**	**6.**	**7.**	**8.**	**9.**	**10.**	**11.**	**12.**	**13.**	**14.**	**15.**	**16.**
1. BMI	−	−	−	−	−	−	−	−	−	−	−	−	−	−	−	−
2. EDE-Q	0.56^∗∗^	−	−	−	−	−	−	−	−	−	−	−	−	−	−	−
3. BAS-RR	–0.09	–0.05	−	−	−	−	−	−	−	−	−	−	−	−	−	−
4. BAS-Drive	–0.03	–0.07	0.54^∗∗^	−	−	−	−	−	−	−	−	−	−	−	−	−
5. BIS	–0.03	0.33^∗∗^	0.29^∗∗^	0.11	−	−	−	−	−	−	−	−	−	−	−	−
6. Reward engagement 250 ms	0.05	0.26^∗∗^	0.09	0.02	–0.03	−	−	−	−	−	−	−	−	−	−	−
7. Reward engagement 500 ms	–0.11	0.02	0.07	–0.04	–0.03	0.34^∗∗^	−	−	−	−	−	−	−	−	−	−
8. Reward disengagement 250 ms	–0.05	–0.02	–0.06	–0.08	0.04	0.00	0.05	−	−	−	−	−	−	−	−	−
9. Reward disengagement 500 ms	0.04	–0.09	0.03	0.04	–0.04	0.10	0.07	0.15	−	−	−	−	−	−	−	−
10. Punishment engagement 250 ms	0.02	–0.17	0.08	0.07	–0.03	–0.34^∗∗^	–0.28^∗∗^	–0.02	–0.16	−	−	−	−	−	−	−
11. Punishment engagement 500 ms	–0.5	0.11	0.04	–0.02	0.11	0.00	–0.04	0.18	0.19	–0.03	−	−	−	−	−	−
12. Punishment disengagement 250 ms	0.12	0.09	–0.03	–0.04	0.00	0.05	0.17	–0.07	–0.22	–0.12	–0.18	−	−	−	−	−
13. Punishment disengagement 500 ms	0.02	0.03	0.20	0.04	–0.01	–0.04	–0.12	–0.27^∗∗^	–0.08	0.16	–0.08	–0.05	−	−	−	−
14. Reward first half	0.31^∗∗^	0.12	–0.08	0.00	0.03	0.05	0.03	–0.05	0.11	0.04	–0.11	–0.04	0.12	−	−	−
15. Reward second half	0.25^∗^	0.09	–0.09	–0.01	0.05	0.01	0.01	–0.10	–0.04	0.06	–0.27^∗∗^	0.00	0.05	0.68^∗∗^	−	−
16. Punishment first half	0.24^∗^	0.04	0.04	0.13	0.06	0.09	0.01	–0.06	0.12	0.11	–0.07	–0.04	0.07	0.87^∗∗^	0.60^∗∗^	−
17. Punishment second half	0.32^∗∗^	0.04	–0.11	–0.04	–0.08	0.10	–0.05	–0.16	–0.01	0.08	−0.23^∗^	0.07	0.08	0.80^∗∗^	0.76^∗∗^	0.74^∗∗^

**^∗^p < 0.05, ^∗∗^p < 0.01.**
